# Relative Brain Perfusion Differences in a Patient With Persistent Fatigue and Subjective Cognitive Symptoms: A Case Report

**DOI:** 10.7759/cureus.118112

**Published:** 2026-10-18

**Authors:** Binh Pham, Penelope Edillor

**Affiliations:** 1 Sleep Medicine, Amen Clinics, Costa Mesa, USA; 2 Psychiatry, Western University of Health Sciences, Pomona, USA

**Keywords:** brain fog, brain perfusion spect, case report, cerebral perfusion, cognitive screening, functional neuroimaging, hyperbaric treatment, persistent fatigue, single-photon emission computed tomography (spect), subjective cognitive symptoms

## Abstract

Persistent fatigue may be accompanied by subjective cognitive symptoms when the available evaluation does not identify a single explanatory disorder. We report the case of a 57-year-old woman with persistent fatigue, reduced mental clarity, and subjective memory difficulties without a loss of independence in daily functioning. Cognitive, mood, attention, and laboratory assessments were performed, although important diagnostic domains remained unassessed or incompletely characterized. Brain perfusion single-photon emission computed tomography (SPECT) was subsequently obtained as an adjunctive functional imaging study. Brain perfusion SPECT depicts relative regional tracer distribution rather than providing an absolute measurement of cerebral blood flow, and the study demonstrated a nonspecific mixed pattern of relative cortical and subcortical tracer distribution. An individualized management plan was subsequently implemented that included a nonstandardized oxygen-enriched hyperbaric intervention using concentrator-supplied chamber air rather than conventional hyperbaric oxygen therapy (HBOT). At approximately 60 days of follow-up, the patient reported improvement in fatigue and exercise tolerance, including the resumption of hiking. However, the improvement was based on patient self-report, standardized pre- and post-treatment outcome measures were not obtained, and additional interventions were recommended concurrently; therefore, the independent contribution of the hyperbaric intervention cannot be determined. This case illustrates the potential descriptive value of adjunctive functional imaging and longitudinal clinical observation in a complex presentation of persistent fatigue and subjective cognitive symptoms while emphasizing that imaging findings and short-term clinical changes should be interpreted within the broader clinical context.

## Introduction

Persistent fatigue is a common and potentially disabling clinical complaint that may substantially affect physical activity, occupational functioning, and the quality of life. Its differential diagnosis is broad and may include medical, sleep-related, psychiatric, medication-related, neurological, autonomic, and postinfectious contributors [1]. Subjective cognitive symptoms, often described as reduced mental clarity, memory difficulty, impaired concentration, or “brain fog,” may accompany persistent fatigue and can occur despite preserved performance on brief cognitive screening. Importantly, subjective cognitive decline is a heterogeneous clinical construct and does not independently establish mild cognitive impairment or an underlying neurodegenerative disorder [2].

Functional neuroimaging has been explored as an adjunctive assessment in patients with persistent neurological and cognitive symptoms. Brain perfusion single-photon emission computed tomography (SPECT) depicts relative regional distribution of an administered technetium-99m (99mTc)-labeled radiotracer rather than providing a direct measurement of absolute cerebral blood flow, and regional differences in tracer distribution are nonspecific [3]. Brain perfusion SPECT has been studied in mild traumatic brain injury, although substantial methodological heterogeneity and uncertainty regarding its incremental diagnostic value remain [4]. Hyperbaric oxygen therapy (HBOT) has also been investigated for persistent symptoms following traumatic brain injury, with inconsistent findings across clinical studies and heterogeneous treatment protocols [5-7]. These bodies of literature provide limited contextual background because the patient reported remote head impacts; however, she did not have an established diagnosis of traumatic brain injury or persistent postconcussion syndrome, and these data should not be directly extrapolated to her presentation.

We present the case of a 57-year-old woman with persistent fatigue and subjective cognitive symptoms who underwent cognitive, mood, attention, laboratory, and adjunctive functional imaging assessment. Important diagnostic domains remained unassessed or incompletely characterized. The available record did not include a documented formal neurological or general physical examination, structural brain magnetic resonance imaging (MRI), objective sleep testing, orthostatic or autonomic assessment, or targeted evaluation for post-COVID-19 condition or myalgic encephalomyelitis/chronic fatigue syndrome. Mild elevations in aspartate aminotransferase, alanine aminotransferase, and ferritin were also not further characterized within the available evaluation. Brain perfusion SPECT demonstrated a heterogeneous pattern of relative cortical and subcortical tracer distribution, but these findings are presented descriptively and cannot establish the cause of the patient’s symptoms. During a subsequent nonstandardized oxygen-enriched hyperbaric intervention, she reported improvement in fatigue and exercise tolerance. The educational value of this case lies in illustrating the interpretive limitations that arise when nonspecific functional imaging findings and short-term clinical changes are observed in the setting of an incomplete diagnostic evaluation and a nonstandardized therapeutic exposure. The purpose of this report is to describe the clinical presentation, available evaluation, functional imaging findings, treatment course, and longitudinal outcome while explicitly acknowledging the unresolved diagnostic limitations and avoiding conclusions regarding etiology, the diagnostic utility of SPECT, or the therapeutic efficacy of the hyperbaric intervention.

## Case presentation

Symptom timeline and clinical history

A 57-year-old woman presented on day 1 with persistent fatigue, low energy, brain fog, and subjective memory difficulties. She reported that the more persistent fatigue syndrome had begun approximately five years before day 1 without an identifiable precipitating event. At later follow-up, however, she recalled similar episodes of pronounced afternoon fatigue approximately three decades earlier and again about a decade later, suggesting that the current syndrome represented a more sustained recurrence rather than her first lifetime episode. Her current symptoms occurred daily with fluctuating severity, typically averaging six to seven out of 10, and became most prominent during the early to mid-afternoon rather than upon awakening. She characterized the sensation primarily as mental fatigue or feeling “tired in the head,” although, on more severe days, it progressed to generalized physical exhaustion that limited walking, exercise, and long-distance driving. Daytime naps provided little relief. She reported a consistent sleep schedule with approximately eight to 10 hours of sleep nightly and denied difficulty with sleep initiation or maintenance.

On day 1, she reported that persistent brain fog and subjective memory difficulties had begun approximately three years earlier. She described reduced mental clarity and difficulty recalling the contents of books she had previously read and television programs she had watched months or years earlier. However, she denied significant difficulty remembering names, conversations, appointments, or routine daily responsibilities; did not frequently misplace belongings; and had not experienced episodes of disorientation or becoming lost. These symptoms had not resulted in the loss of independence in daily functioning, although she expressed concern about their potential impact on returning to work and maintaining her caregiving responsibilities. Her concern regarding cognitive decline was heightened by a maternal family history of dementia involving both her mother and grandmother.

Her history was notable for several remote head impacts without a reported loss of consciousness or persistent neurological sequelae. In early adulthood, she sustained a posterior head impact after falling backward onto concrete. Years later, she experienced whiplash during a motor vehicle accident and, on another occasion, was struck on the frontal aspect of the head by a sailing boom. She denied lasting neurological symptoms following these events. She denied heavy snoring, known sleep apnea, sleepwalking, or sleep bruxism. She also denied depressed mood, anhedonia, hopelessness, worthlessness, excessive guilt, appetite disturbance, psychomotor slowing, or suicidal ideation.

Cognitive, mood, and attention assessment

Neuropsychiatric assessment included brief cognitive, mood, and attention screening. The Self-Administered Gerocognitive Examination (SAGE) score was 20/22, within the reported normal range [8]. The Patient Health Questionnaire-9 (PHQ-9) score was 2/27, consistent with minimal depressive symptoms [9]. Computerized testing with the Conners Continuous Performance Test (CPT) did not suggest a disorder characterized by attention deficits [10]. Formal neuropsychological testing was not performed.

Important diagnostic domains were not represented in the available retrospective record. Specifically, the record did not include a documented formal neurological or general physical examination, structural brain magnetic resonance imaging, objective sleep testing, orthostatic or autonomic assessment, or targeted evaluation for post-COVID-19 condition or myalgic encephalomyelitis/chronic fatigue syndrome. These unassessed domains limited the characterization of potential contributors to the patient’s symptoms.

Laboratory workup

Laboratory testing available during the clinical evaluation assessed several potentially reversible contributors to the patient’s persistent fatigue and subjective cognitive symptoms (Table 1). Complete blood count, renal function, electrolytes, serum glucose, thyroid studies, vitamin D, and high-sensitivity C-reactive protein were within the performing laboratory’s reference ranges. Mild elevations in aspartate aminotransferase (42 IU/L), alanine aminotransferase (57 IU/L), and ferritin (206 ng/mL) were noted. Hemoglobin A1c was 4.7%, slightly below the performing laboratory’s reference interval. Follicle-stimulating hormone (FSH) and estradiol values fell within the laboratory’s reported postmenopausal reference ranges. The elevations in aminotransferases and ferritin were not further characterized within the available evaluation. These findings did not identify a single laboratory abnormality that adequately explained the clinical presentation.

**Table 1:** Selected laboratory evaluation. Selected laboratory evaluation performed during the clinical assessment. No single hematologic, metabolic, thyroid, inflammatory, or reproductive-hormone abnormality clearly explained the patient’s persistent fatigue and subjective cognitive symptoms. Mild elevations in AST, ALT, and ferritin and a slightly low hemoglobin A1c were noted. FSH and estradiol values fell within the performing laboratory’s reported postmenopausal reference ranges. Reference ranges are those reported by the performing laboratory. 25-OH: 25-hydroxy

Laboratory test	Result	Reference range
White blood cell count	6.9 × 10³/µL	3.4-10.8
Hemoglobin	13.2 g/dL	11.1-15.9
Creatinine	0.80 mg/dL	0.57-1.00
Glucose	82 mg/dL	70-99
Aspartate aminotransferase (AST)	42 IU/L	0-40
Alanine aminotransferase (ALT)	57 IU/L	0-32
Hemoglobin A1c	4.70%	4.8-5.6
Thyroid-stimulating hormone (TSH)	0.846 µIU/mL	0.450-4.500
Free thyroxine (free T4)	1.08 ng/dL	0.82-1.77
Free triiodothyronine (free T3)	2.4 pg/mL	2.0-4.4
Follicle-stimulating hormone (FSH)	57.5 mIU/mL	Postmenopausal: 25.8-134.8
Estradiol	37.3 pg/mL	Postmenopausal: <6.0-54.7
Vitamin D (25-OH)	76.8 ng/mL	30.0-100.0
High-sensitivity C-reactive protein	0.25 mg/L	0.00-3.00
Ferritin	206 ng/mL	15-150

Subsequent laboratory evaluation further assessed potential infectious contributors to the persistent symptoms. Serologic and molecular testing included evaluation for Epstein-Barr virus (EBV), cytomegalovirus (CMV), human herpesvirus 6 (HHV-6), and Lyme disease (Table 2).

**Table 2:** Infectious serologic and molecular testing. Infectious serologic and molecular testing obtained during the clinical evaluation. EBV VCA IgG was elevated with negative VCA IgM, a pattern that did not support acute primary EBV infection. EBV nuclear antigen serology was not available; therefore, complete EBV serologic staging could not be performed. The remaining infectious studies were negative. These findings did not establish an active infectious explanation for the patient’s persistent fatigue and subjective cognitive symptoms. EBV, Epstein-Barr virus; VCA, viral capsid antigen; IgG, immunoglobulin G; IgM, immunoglobulin M; CMV, cytomegalovirus; HHV-6, human herpesvirus 6; PCR, polymerase chain reaction

Test	Result	Interpretation
EBV VCA IgG	595 U/mL, elevated	Supports prior EBV exposure; the magnitude of IgG elevation alone does not establish recent infection or reactivation
EBV VCA IgM	<36 U/mL, negative	Does not support acute primary EBV infection
CMV IgG	Negative	No detectable CMV IgG
CMV IgM	Negative	Does not support acute CMV infection
HHV-6 PCR	Negative	HHV-6 DNA was not detected
Lyme disease IgG immunoblot	Negative	No serologic evidence of Lyme disease by the reported immunoblot
Lyme disease IgM immunoblot	Negative	No serologic evidence of Lyme disease by the reported immunoblot

Overall, the infectious evaluation did not establish an active infectious process accounting for the patient’s persistent symptoms. The elevated EBV viral capsid antigen (VCA) immunoglobulin G (IgG) was interpreted as evidence of EBV exposure rather than evidence of acute infection, reactivation, or a causal relationship with the clinical presentation; because EBV nuclear antigen serology was unavailable, complete serologic staging could not be performed. Given her subjective memory concerns and maternal family history of dementia, plasma phosphorylated tau 217 (p-tau217) was also obtained. The concentration was 0.10 pg/mL, within the performing laboratory’s reference interval of 0.00-0.18 pg/mL. This result was considered, alongside the clinical and cognitive assessment, and was not interpreted independently as establishing or excluding a neurodegenerative disorder.

SPECT protocol and findings

Following the initial clinical and laboratory evaluation, brain perfusion single-photon emission computed tomography (SPECT) was obtained at Amen Clinics as an adjunctive functional imaging study. The clinical SPECT documentation identified technetium-99m hexamethylpropyleneamine oxime (99mTc-HMPAO) as the radiopharmaceutical. Resting and concentration studies were obtained on separate days.

For the resting study, the patient was placed in a dimly lit, quiet room and remained quiet with her eyes open for several minutes before radiotracer administration. During the concentration study, following a similar initial equilibration period, she performed the Conners Continuous Performance Test, a 15-minute computerized sustained-attention task [10]. Radiotracer was administered approximately three minutes after the initiation of the task, after which she completed the CPT. The CPT served as a standardized sustained-attention condition during the concentration study rather than as a standalone diagnostic assessment of her cognitive symptoms. The paired studies were interpreted descriptively and were not considered quantitative measurements of cerebral activation or absolute cerebral blood flow.

According to the clinical SPECT documentation, tomographic imaging was performed approximately 30-60 minutes after radiotracer administration using high-resolution gamma-camera imaging with fan-beam collimation. Data were acquired using a 128 × 128 matrix with 120 projections obtained at 3° intervals over 360°. The data were prefiltered using a low-pass filter with a high cutoff, and linear attenuation correction was applied. Images were reconstructed and reviewed in coronal, sagittal, and transaxial planes and as three-dimensional renderings. Tomographs were displayed using a standardized linear color scale. Visual interpretation incorporated three-dimensional surface maps displaying regions with the highest 45% of relative tracer activity and three-dimensional active maps comparing average relative tracer activity with the highest 15% of relative tracer activity. The patient-specific administered radiotracer activity and exact imaging system were not available for retrospective verification; therefore, technical details are presented only as documented in the available clinical imaging record.

According to the qualitative clinical SPECT interpretation, the resting and concentration studies were described as demonstrating a heterogeneous pattern of relative regional tracer distribution involving cortical and subcortical structures. Relative decreases were described in the medial prefrontal pole, medial dorsal prefrontal cortex, bilateral temporal regions, left parietal region, cerebellum, and regions along the longitudinal fissure on both studies, with additional bilateral inferior orbitofrontal decreases described at rest. The right temporal region was described as demonstrating a mixed pattern, with areas of mildly increased tracer activity, as well as areas of relatively decreased activity. Relatively increased tracer activity was also described in the bilateral basal ganglia and focal thalamic regions on both studies. These observations were based on the qualitative clinical interpretation; validated quantitative regional analysis, standardized normative comparator maps, validated quantitative between-session analysis, and formal inter-reader assessment were not available for retrospective review. The imaging findings were interpreted descriptively within the broader clinical context and were not considered independently diagnostic of traumatic brain injury, a neurodegenerative disorder, or another specific neurological or psychiatric condition. Representative surface-rendered resting SPECT images are shown in Figure 1, and representative subcortical resting SPECT images are shown in Figure 2.

**Figure 1:**
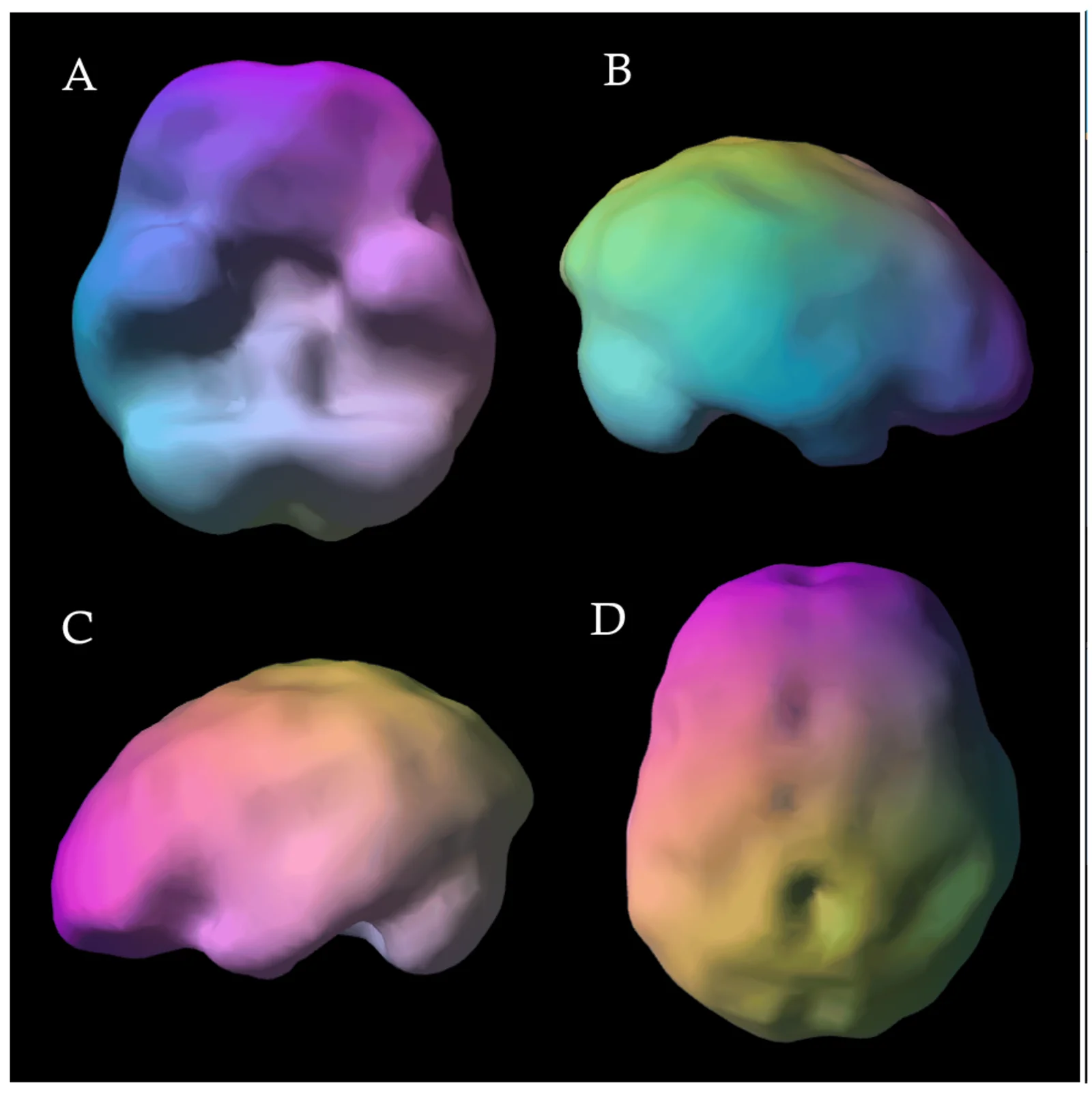
Surface-rendered resting brain perfusion SPECT images. Surface-rendered brain perfusion single-photon emission computed tomography (SPECT) images demonstrating the relative regional distribution of tracer activity during the resting study. (A) Inferior (underside) view. (B) Right lateral view. (C) Left lateral view. (D) Superior (top-down) view. The clinical interpretation describes relatively decreased tracer activity involving the bilateral inferior orbitofrontal cortex, medial prefrontal pole and medial dorsal prefrontal regions, bilateral temporal regions, left parietal region, and cerebellum. The right temporal region demonstrated a heterogeneous pattern with areas of relatively greater and relatively lower tracer activity. The color-rendered images represent relative within-patient tracer intensity and do not provide direct or absolute measurements of cerebral blood flow. Surface rendering was generated from the relative regional tracer distribution according to the clinical imaging protocol. These images are presented for descriptive and illustrative purposes and are not independently diagnostic of traumatic brain injury, neurodegenerative disease, or another specific neurological or psychiatric disorder. SPECT: single-photon emission computed tomography

**Figure 2:**
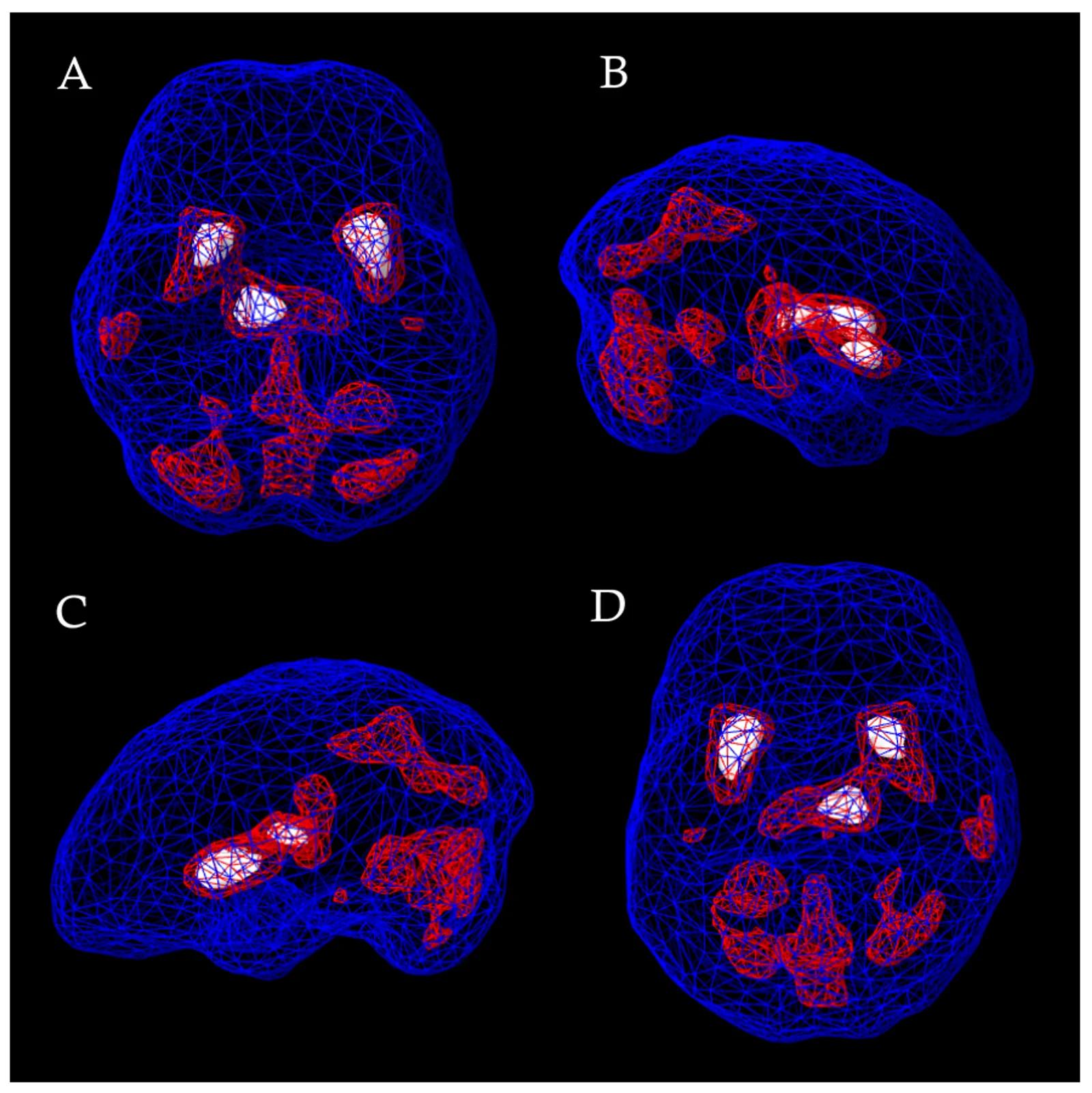
Subcortical resting brain perfusion SPECT images. Subcortical brain perfusion single-photon emission computed tomography (SPECT) images demonstrating the relative regional distribution of tracer activity during the resting study. (A) Inferior (underside) view. (B) Right lateral view. (C) Left lateral view. (D) Superior (top-down) view. The clinical interpretation describes relatively increased tracer activity involving the bilateral basal ganglia and focal thalamic regions. Within the three-dimensional active rendering, red-to-white regions represent relatively greater tracer activity, while the blue wireframe provides an anatomical surface reference. The displayed activity represents relative within-patient tracer distribution and does not provide direct or absolute measurements of cerebral blood flow. These images are presented for descriptive and illustrative purposes and are not independently diagnostic of traumatic brain injury, neurodegenerative disease, or another specific neurological or psychiatric disorder. SPECT: single-photon emission computed tomography

Nonstandardized oxygen-enriched hyperbaric intervention and follow-up

Following the initial clinical evaluation, an individualized management plan was implemented that included a nonstandardized oxygen-enriched hyperbaric intervention, together with general recommendations regarding nutrition, sleep optimization, physical activity, and other supportive measures. The intervention was initiated shortly after day 1. By approximately day 60, the patient had completed approximately 15 sessions, generally administered one to two times weekly, with most sessions occurring twice weekly. Each session lasted approximately 60 minutes. Treatment pressure was initiated at 1.5 atmospheres absolute (ATA), subsequently increased to 2.0 ATA, and later advanced to 2.2 ATA as tolerated.

The intervention was administered at Amen Clinics using oxygen-enriched chamber air supplied by an oxygen concentrator rather than a fixed near-100% oxygen exposure. According to treatment staff, chamber oxygen concentration was approximately 75%-80% at pressures up to approximately 1.75 ATA. At higher pressures, increased backpressure on the oxygen supply line was reported to reduce oxygen delivery, and the corresponding chamber oxygen concentration was not quantified. No scheduled air breaks were used. The treatment course included intermittent scheduling breaks, but no sessions were terminated early, no pressure reductions were required for tolerance, and no significant treatment-related adverse effects were reported, including during treatment at 2.2 ATA. The specific chamber model, device regulatory status, and chamber-specific fire-safety safeguards were not available for retrospective verification.

At approximately day 60, the patient reported improvement in fatigue and exercise tolerance, including the resumption of hiking activities that she had been unable to sustain for several years because of fatigue. Sleep remained satisfactory. The reported improvement was based on patient self-report and was not corroborated by validated fatigue scales, objective exercise testing, formal neuropsychological reassessment, or other standardized pre- and post-treatment outcome measures. Because the oxygen-enriched hyperbaric intervention occurred within a broader individualized management plan and adherence to other recommended interventions was not systematically quantified, the independent contribution of the intervention to the observed improvement cannot be determined.

The major clinical events are summarized in Table 3 in accordance with the Case Report (CARE) framework.

**Table 3:** Clinical timeline. Summary of the major historical, diagnostic, treatment, and follow-up events. Day 1 represents the initial clinical evaluation. Historical intervals are approximate and are based partly on retrospective patient report. SPECT: single-photon emission computed tomography

Time relative to day 1	Clinical event
Approximately three decades before day 1	Earlier episode of pronounced afternoon fatigue recalled retrospectively
Approximately two decades before day 1	Additional similar episode of afternoon fatigue recalled retrospectively
Approximately five years before day 1	More persistent fatigue syndrome developed without an identifiable precipitating event
Approximately three years before day 1	Persistent brain fog and subjective memory difficulties developed
Day 1	Initial evaluation for persistent fatigue, low energy, brain fog, and subjective memory concerns
Following day 1	Cognitive, mood, attention, laboratory, infectious, and adjunctive brain perfusion SPECT assessments were completed as described above
Shortly after day 1	Nonstandardized oxygen-enriched hyperbaric intervention was initiated as one component of a broader individualized management plan
Approximately day 60	After approximately 15 sessions, the patient reported improvement in fatigue and exercise tolerance, including the resumption of hiking. Improvement was self-reported; standardized pre- and post-treatment outcome measures were not obtained

## Discussion

This case illustrates the interpretive challenges associated with persistent fatigue and subjective cognitive symptoms when the available evaluation is incomplete and adjunctive functional imaging demonstrates nonspecific findings. Although the patient described substantial mental fatigue, reduced mental clarity, and subjective memory difficulty, brief cognitive screening remained within the reported normal range, depressive symptom burden was minimal, and computerized continuous performance testing did not suggest a disorder characterized by attention deficits [8-10]. Subjective cognitive symptoms may occur despite preserved performance on brief cognitive measures and do not independently establish mild cognitive impairment or an underlying neurodegenerative disorder [2]. Plasma p-tau217 was also within the performing laboratory’s reference interval; however, this finding should be interpreted cautiously. Current clinical guidance for Alzheimer’s disease blood-based biomarkers is directed primarily toward patients with objective cognitive impairment undergoing specialized diagnostic evaluation and does not extend to individuals with subjective cognitive decline alone [11]. Accordingly, the p-tau217 result is best regarded as an additional clinical data point rather than as evidence excluding an underlying neurodegenerative process.

Available laboratory testing identified several mild abnormalities but did not establish a single laboratory explanation for the symptom complex. Aspartate aminotransferase and alanine aminotransferase were mildly elevated, ferritin was elevated, and hemoglobin A1c was slightly below the performing laboratory’s reference interval. These abnormalities were not further characterized within the available evaluation. Other measured hematologic, renal, glucose, thyroid, inflammatory, and vitamin D parameters did not identify a clear explanatory abnormality. Infectious testing likewise did not establish an active infectious process. Epstein-Barr virus viral capsid antigen IgG was markedly elevated, whereas viral capsid antigen immunoglobulin M (IgM) was negative. Viral capsid antigen IgG typically persists following EBV infection, and the magnitude of an IgG elevation alone does not establish recent infection, viral reactivation, or a causal relationship with persistent symptoms [12]. Because EBV nuclear antigen serology was unavailable, complete serologic staging could not be performed. Accordingly, the EBV findings document EBV exposure but, without EBV nuclear antigen serology, do not establish the timing of infection, viral reactivation, or a causal relationship with the patient’s persistent fatigue.

The patient’s remote head impacts represent a contextual factor but do not establish a traumatic etiology for either her current symptoms or the observed SPECT findings. She did not report a loss of consciousness or persistent neurological sequelae following these events, and the more sustained fatigue syndrome emerged many years later. Brain perfusion SPECT has been investigated in mild traumatic brain injury, with frontal and temporal perfusion abnormalities among patterns reported in the literature; however, substantial methodological heterogeneity remains, and the incremental diagnostic value of SPECT is uncertain [4]. Brain perfusion SPECT also depicts relative regional radiotracer distribution rather than a direct measurement of absolute cerebral blood flow [3]. The heterogeneous cortical and subcortical tracer-distribution pattern observed in this patient therefore cannot establish the presence, timing, mechanism, or clinical significance of a previous brain injury and is best interpreted as a descriptive functional imaging finding within the broader clinical context.

Hyperbaric oxygen therapy (HBOT) has been investigated for persistent neurological and cognitive symptoms following traumatic brain injury, although the available evidence remains inconsistent. Wolf et al. did not demonstrate a clear HBOT-specific benefit in a randomized sham-controlled trial of patients with persistent symptoms following mild traumatic brain injury [5]. In contrast, Boussi-Gross et al. reported improvements in cognitive performance, quality of life, and regional brain perfusion following HBOT in patients with chronic postconcussion symptoms [6]. A more recent systematic review and meta-analysis identified potential improvements across several neurocognitive domains while emphasizing the limited number of studies and heterogeneity of treatment protocols [7]. These populations are not directly comparable to the present patient, who did not have an established diagnosis of traumatic brain injury or persistent postconcussion syndrome.

Importantly, the intervention used in this case did not meet the Undersea and Hyperbaric Medical Society definition of conventional HBOT, which requires breathing near-100% oxygen, defined as at least 95% medical-grade oxygen, under increased ambient pressure [13]. The patient instead received a nonstandardized oxygen-enriched hyperbaric intervention using concentrator-supplied chamber air. According to treatment staff, chamber oxygen concentration was approximately 75%-80% at pressures up to approximately 1.75 ATA, while oxygen concentration at higher treatment pressures was not measured. The specific chamber model, device regulatory status, and chamber-specific fire-safety safeguards were not available for retrospective verification. The delivered oxygen dose therefore cannot be precisely characterized. Accordingly, this intervention should not be considered equivalent to conventional HBOT, and findings from published HBOT studies cannot be directly extrapolated to the exposure used in this case.

Biological mechanisms proposed for conventional HBOT include effects on mitochondrial function, neurogenesis, angiogenesis, synaptic and axonal plasticity, and inflammatory pathways [14]. In a small clinical study of patients with chronic traumatic brain injury, Tal et al. reported interval changes in cerebral perfusion and diffusion tensor imaging measures following HBOT that were associated with cognitive improvement [15]. These observations remain hypothesis-generating and cannot be assumed to apply to the present patient because her oxygen exposure differed substantially from conventional HBOT protocols. No corresponding molecular, physiological, or longitudinal neuroimaging measurements were obtained in this patient. A biological mechanism linking the oxygen-enriched hyperbaric intervention to her subsequent clinical course therefore cannot be established.

In the present patient, the nonstandardized oxygen-enriched hyperbaric intervention was initiated at 1.5 ATA and progressively increased to 2.2 ATA as one component of a broader individualized management plan. During the treatment period, she reported improvement in fatigue and exercise tolerance, including the resumption of hiking activities that she had been unable to sustain for several years. Recommendations regarding nutrition, sleep optimization, physical activity, and other supportive measures were provided during the same period, and adherence to individual components of the management plan was not systematically quantified. The reported improvement therefore represents a temporal observation during a multimodal treatment period and does not establish therapeutic efficacy, a dose-response relationship, or an independent effect of the hyperbaric intervention. Taken together, this case illustrates the potential descriptive value of adjunctive functional imaging and longitudinal clinical observation in complex presentations of persistent fatigue and subjective cognitive symptoms while emphasizing that these findings are most informative when integrated with the broader clinical evaluation rather than interpreted in isolation.

Several limitations relate to the characterization of the clinical syndrome and its potential contributors. Portions of the history were retrospective, including remote head impacts and earlier episodes of fatigue for which contemporaneous records were unavailable. Formal neuropsychological testing was not performed, and preserved performance on brief cognitive and attention screening does not exclude subtle domain-specific deficits. Importantly, the available record did not include a documented formal neurological or general physical examination, structural brain MRI, objective sleep testing, orthostatic or autonomic assessment, or targeted evaluation for post-COVID-19 condition or myalgic encephalomyelitis/chronic fatigue syndrome. The mild elevations in aminotransferases and ferritin were also not further evaluated within the available record. These unresolved diagnostic gaps materially limit the etiologic interpretation of the patient’s symptoms and the clinical context in which the SPECT findings can be interpreted. Although FSH and estradiol values fell within the performing laboratory’s reported postmenopausal reference ranges, the potential contribution of the menopausal transition was also not fully characterized. On day 1, the patient reported that persistent brain fog had begun approximately three years earlier, but the timing of her final menstrual period, presence and severity of vasomotor or other menopausal symptoms, use of hormone therapy, and temporal relationship between these factors and symptom onset were not systematically documented. Menopause-related factors therefore remain a potential but unconfirmed contributor to the clinical presentation.

Additional limitations relate to the imaging and characterization of the hyperbaric exposure. The SPECT studies were obtained as part of routine clinical care and depict relative regional tracer distribution rather than quantitative measurements of absolute cerebral blood flow. The exact imaging system and patient-specific administered radiotracer activity were unavailable for retrospective verification, validated quantitative regional analysis and standardized normative comparator maps were unavailable, and no longitudinal post-treatment SPECT study was obtained to determine whether the imaging pattern changed over time. The oxygen-enriched hyperbaric exposure was also incompletely characterized. Chamber oxygen concentration was estimated only at lower treatment pressures and was not measured at the higher pressures used later in treatment. The specific chamber model, regulatory status, and chamber-specific fire-safety safeguards were unavailable for retrospective verification. These limitations prevent the precise characterization of the delivered oxygen exposure and substantially limit comparison with established HBOT protocols.

The interpretation of the clinical outcome is also substantially limited by the nature and duration of follow-up. Improvement in fatigue and exercise tolerance was based solely on patient self-report over approximately 60 days and was not corroborated by validated symptom scales, objective exercise testing, formal neuropsychological assessment, or other standardized pre- and post-treatment outcome measures. The oxygen-enriched hyperbaric intervention occurred within a broader individualized management plan that included concurrent recommendations regarding nutrition, sleep optimization, physical activity, and other supportive measures, and adherence to these individual interventions was not systematically quantified. In the absence of standardized baseline and follow-up measures, longer-term observation, or a comparator condition, natural symptom fluctuation, nonspecific treatment effects, concurrent interventions, and other unmeasured factors cannot be distinguished from any potential effect of the hyperbaric exposure. Accordingly, these observations do not establish the diagnostic utility of brain perfusion SPECT, the therapeutic efficacy of the hyperbaric intervention, the durability of the reported improvement, or an independent treatment effect. Prospective studies incorporating standardized clinical outcomes, quantitative neuroimaging methods, and clearly defined hyperbaric treatment protocols are needed to clarify the potential roles of functional neuroimaging and hyperbaric interventions in patients with persistent fatigue and cognitive symptoms.

## Conclusions

Persistent fatigue and subjective cognitive symptoms may remain clinically challenging when the available evaluation does not identify a single explanatory disorder. This case documents a heterogeneous pattern of relative cortical and subcortical tracer distribution on brain perfusion SPECT in a patient who subsequently reported improved fatigue and exercise tolerance during a broader management course that included a nonstandardized oxygen-enriched hyperbaric intervention. Although the relationship between the imaging findings, symptoms, and subsequent improvement cannot be established from a single case, the report illustrates the potential descriptive value of adjunctive functional imaging and careful longitudinal observation in clinically complex presentations while emphasizing the need to interpret both imaging findings and treatment response within the broader diagnostic context.
